# Lingual Frenotomy in Breastfeeding Infants: An Umbrella Review

**DOI:** 10.1111/ipd.70031

**Published:** 2025-09-24

**Authors:** Bruno Valério da Silva, Amanda Vieira Barollo, Normanda da Nóbrega Lima Sá, Luciana Butini Oliveira, Luciana Faria Sanglard

**Affiliations:** ^1^ Federal University of Espírito Santo Vitória Brazil; ^2^ Faculdade São Leopoldo Mandic Campinas Brazil

**Keywords:** ankyloglossia, infant, lingual frenotomy

## Abstract

**Background:**

Clinical diagnosis of ankyloglossia and its therapeutic management through lingual frenotomy (LF) have increased in several countries. However, systematic reviews (SRs) and experts opinions show conflicting results on this topic.

**Aim:**

To synthesize the available knowledge with a critical evaluation of SRs on LF in infants up to 1 year of age on breastfeeding‐related outcomes.

**Design:**

An umbrella review was carried out. Searches were conducted (seven electronic databases, gray literature). SRs of clinical trials involving infants up to 1 year of age undergoing LF to treat maternal self‐efficacy related to breastfeeding, nipple pain, and/or weight gain were included. The articles were critically analyzed (AMSTAR 2).

**Results:**

We identified 272 studies; 15 SRs were included. Confidence was classified as critically low (*n* = 14; 93.3%) and moderate (*n* = 1; 6.7%). Fourteen SRs had more than two critical aspects, indicating that the studies were not conducted properly.

**Conclusion:**

The evidence on the efficacy of LF in infants remains inconclusive in relation to breastfeeding outcomes, especially maternal self‐efficacy related to breastfeeding and weight gain. Evidence of benefit for the treatment of breast pain is weak. Overdiagnosis of ankyloglossia may lead to overtreatment of the condition, without high‐quality evidence to support the benefits of this procedure.

**Summary**

- Why this paper is important to pediatric dentists
  ○ This study provides a critical review of the evidence for the efficacy of LF on breastfeeding outcomes (self‐efficacy and nipple pain) and weight gain in infants up to one year of age.
  ○ Given the substantial heterogeneity across studies and the predominantly critically low methodological quality assessed through the Amstar 2 tool, the current evidence remains inconclusive. These findings underscore the need for cautious interpretation and highlight the importance and improving the methodological rigor of future studies.
  ○ Pediatric dentists and other healthcare professionals should remain prudent in their clinical decision‐making, avoiding premature conclusions and being aware of the potential risks associated with overdiagnosis and overtreatment of ankyloglossia.

## 1 Introduction

Ankyloglossia causes restricted tongue movement due to shortening, thickening, or tightening of the lingual frenulum [1, 2]. Breastfeeding, swallowing, and speech can be affected by functional problems caused by these alterations. However, the impact of this association is still unknown [1, 2, 3].

Data on the prevalence of ankyloglossia vary widely because many different diagnostic tools have been used: Coryllos: 20%; Hazelbaker Assessment Tool for Lingual Frenulum Function (HATLFF) associated with Coryllos: 18%; Kotlow: 9%; Bristol Tongue Assessment Tool (BTAT): 11%; Neonatal Tongue Screening Test: 9%; non‐specific tools: 2%; or age range—breastfeeding infants: 7%; children: 1%; and adolescents: 2% [1]. In the United States, between 1997 and 2013, diagnosis of ankyloglossia and completion of lingual frenotomy (LF) increased by 834% and 866%, respectively. The majority of patients were less than 1 year old (98.8%). Frenotomy/frenulotomy is a simple cut or incision of the frenulum used to treat ankyloglossia [4]. This scenario has caused concern among medical and dental associations and demonstrates the need for a careful, multidisciplinary approach to diagnosis and treatment [5].

There are overviews of this issue. Power and Murphy (2015) [6] in their study, restricted the literature search and did not carry out a critical appraisal of the systematic reviews (SR) included in their review. In addition, they included other study designs. Butenko et al. (2019) [7] conducted their review by limiting the date range of the included publications and although studies were not excluded based on study design, the highest level of evidence was sought [8].

Systematic reviews of high methodological quality are very important for decision‐making in clinical practice. Well‐conducted SRs are considered the highest level of evidence and can help guide clinical practice or even signal a lack of supporting evidence [9]. From this perspective, AMSTAR 2 is used to assess the methodological characteristics of SRs [10].

This study aimed to synthesize available information on the impact of infant LF on breastfeeding‐related outcomes. An umbrella review was completed to provide a critical appraisal of SRs on this subject.

## 2 Materials and Methods

**FIGURE 1:**
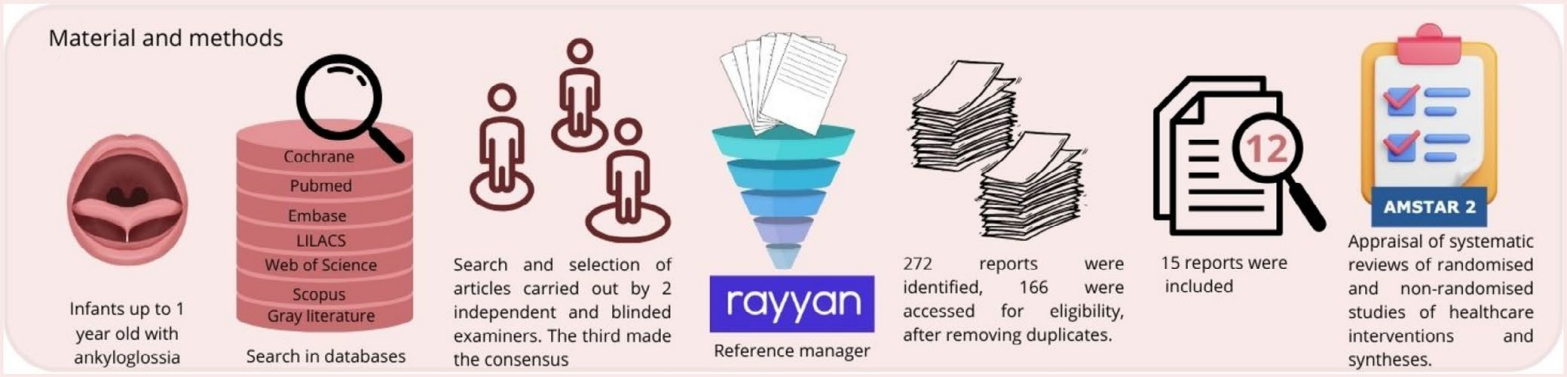
Summary scheme of materials and methods.

A summary of the materials and methods is shown in Figure 1.

### 2.1 Research Question

The research question followed the PICO structure (*P* = breastfeeding infants up to 1 year old; I = lingual frenotomy; C = other interventions or none; O = maternal self‐efficacy, maternal nipple pain, and weight gain), to answer the following question: What do we know, from SRs, about the available evidence on the results of frenotomy on infant breastfeeding outcomes?

### 2.2 Criteria for Eligibility

Inclusion criteria were as follows: (1) SRs of clinical trials, with or without meta‐analysis; (2) that addressed LF performed on infants up to 1 year of age; (3) studies that evaluated outcomes related to breastfeeding, such as maternal self‐efficacy, nipple pain, and/or weight gain.

Exclusion criteria were as follows: (1) SRs that included populations other than babies up to 1 year old, and (2) narrative or integrative reviews, observational studies, in vitro laboratory research, observational, prognostic, diagnostic, qualitative and mixed methods, methodological and cost‐effectiveness analyses, abstracts, commentaries, case reports, protocols, expert opinions, and letters to the editor and posters. There were no restrictions on language or year of publication.

### 2.3 Information Sources and Search Strategies

The databases used were the Cochrane Library, Embase, LILACS, PubMed, Scopus, Web of Science, and Google Scholar. The search strategy (Appendix S1) was reviewed and refined by a librarian for each database. The initial bibliographic search was carried out on February 6, 2024, and updated on December 18, 2024.

### 2.4 Data Selection and Collection Process

The studies retrieved from the databases were exported to Rayyan for reference management [11]. Duplicate studies were excluded. Two independent reviewers (NLS and BVS) retrieved the studies, screened, and read the full texts to confirm eligibility. A third reviewer was responsible for consensus (LFS) in case of disagreement. A manual search was also carried out on the references of the included articles.

### 2.5 Data Items

The variables (authors, year, country, registry, presence of meta‐analysis, main results and conclusions) were extracted by two independent reviewers (AVB and BVS), with a third responsible for consensus (LFS) in case of disagreement.

### 2.6 Assessing the Risk of Bias

The methodological quality of the included studies was assessed by two independent reviewers (AVB e BV) using AMSTAR 2 (A MeaSurement Tool to Assess Systematic Reviews) [12]. The tool provides 16 checklist items, of which seven are critical (2, 4, 7, 9, 11, 13, and 15) and nine non‐critical (1, 3, 5, 6, 8, 10, 12, 14, and 16). AMSTAR 2 classifies overall confidence in the results of the review as: (1) High, in cases of zero or one non‐critical weakness; (2) Moderate, more than one non‐critical weakness; (3) Low, one critical failure with or without non‐critical weaknesses; and (4) Critically low, more than one critical failure with or without non‐critical weaknesses [12].

## 3 Results

### 3.1 Systematic Review and Selection of Supplementary Primary Studies

**FIGURE 2:**
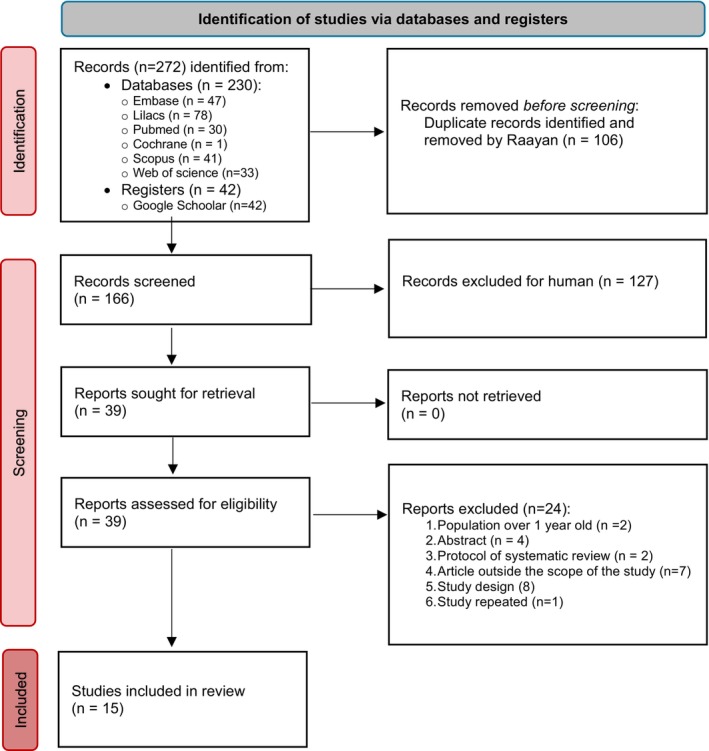
Flowchart that includes searches of databases and registers.

The searches were carried out on PubMed/Medline (*n* = 30), Embase (*n* = 47), Scopus (*n* = 41), Web of Science (*n* = 33), Cochrane (*n* = 1), LILACS (*n* = 78), and Google Scholar (*n* = 42), resulting in a total of 272 references (Figure 2).

After removing duplicates, the titles and abstracts of 166 studies were analyzed and selected according to the eligibility criteria. Thirty‐nine studies were read in full, and 15 were included (Figure 2). The reasons for excluding 24 studies can be found in Appendix S2.

### 3.2 Characteristics of Systematic Reviews and Supplemental Primary Studies

**FIGURE 3:**
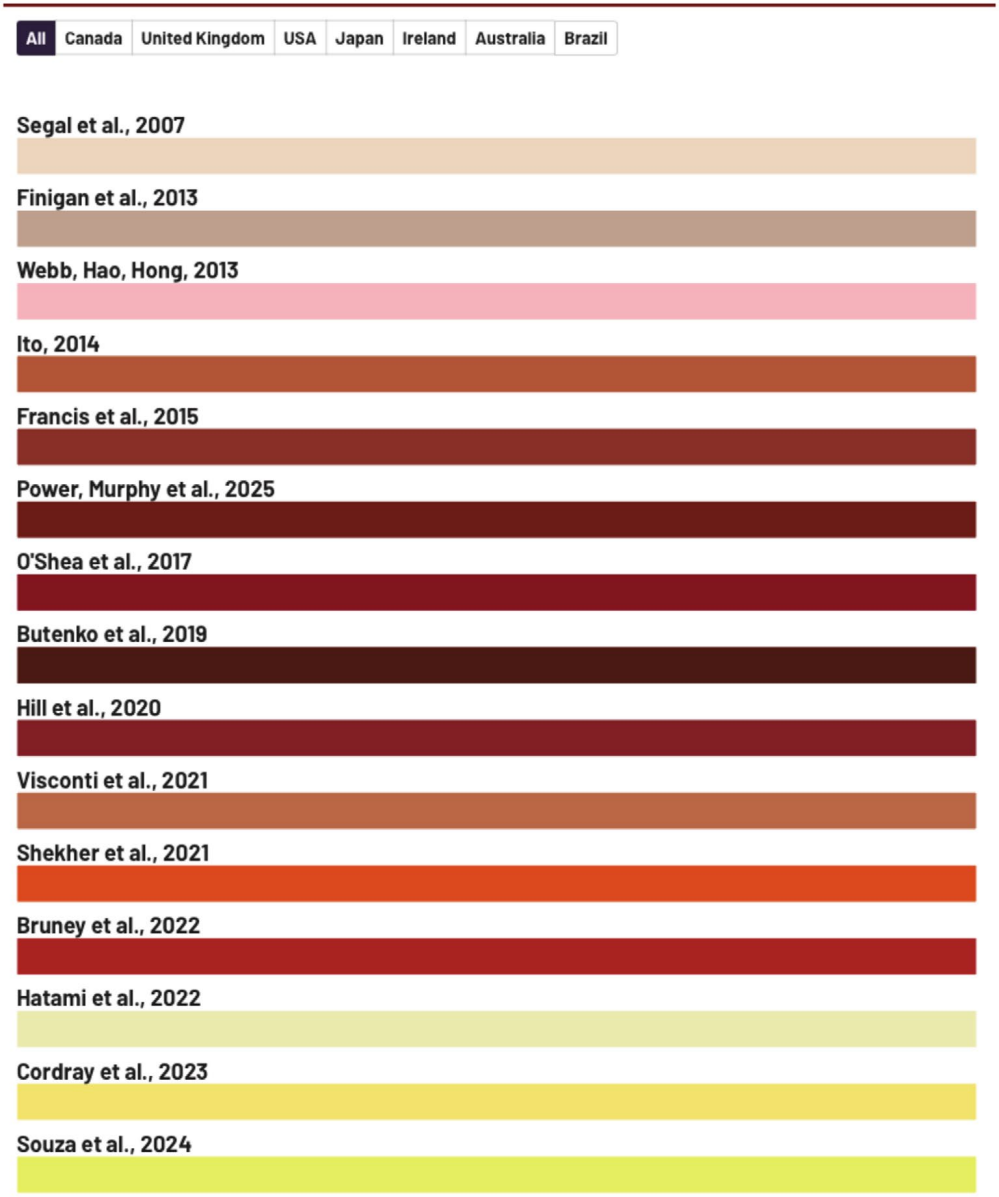
Characteristics of the systematic reviews included in the study. An interactive version of this treemap is available online: https://flourish‐user‐preview.com/api/canva/embed/visualisation/20241976/THGWhpsPDQwsgKPfN2KeuW8GTC2fpQQcjV0ei_‐qVIqKtecZ38MMi8OHTWqLoT90/.

A treemap with the data of the included SRs can be found in Figure 3. An interactive version of this treemap is available online: https://flourish‐user‐preview.com/api/canva/embed/visualisation/20241976/THGWhpsPDQwsgKPfN2KeuW8GTC2fpQQcjV0ei_‐qVIqKtecZ38MMi8OHTWqLoT90/. The overview included 15 studies that addressed outcomes related to pain, breastfeeding difficulties, and/or infant weight gain. Of these, only two had protocols registered prior to publication [13, 14]. Five SRs conducted meta‐analyses [10, 13, 14, 15, 16], whereas one was a scoping review [17] and another an overview [2]. The scoping review by Souza et al. [17] included 46 studies, but only one was a randomized controlled trial. As clinical trials are the most appropriate design to evaluate the efficacy of frenotomy, the predominance of observational studies in this review limits the strength of the evidence on this intervention. The review by Butenko et al. [7] included three SRs [10, 18, 19]. One of these was a book chapter published by the Canadian Agency for Drugs and Technologies in Health and was excluded from our analysis [18].

Regarding geographical distribution, the majority of reviews (46.6%, *n* = 7) were led by corresponding authors based in the United States [10, 14, 16, 19, 20, 21, 22]. The remaining studies were from Canada [13, 14], the United Kingdom [15], Japan [10], Australia [7, 23], Brazil [17], and Ireland [6].

Two reviews had broader age ranges: one included children from 0 to 12 years [19] and another from 0 to 18 years [14]. In both cases, it was possible to extract and analyze data specific to infants aged 0 to 1 year. Additionally, to avoid duplication, the study by Francis et al. (2015) [24] was excluded, as it presented the same data already reported in Francis et al. (2015) [22] (Figure 3).

The SRs included a wide variety of primary studies with different research designs. Segal et al. (2007) [25] used a randomized clinical trial (RCT), prospective trials, case series, cohort studies, controlled studies, and case reports. Finigan et al. (2013) [26] included four RCTs, case studies, prospective studies, and a SR. This SR [26] was identified and critically analyzed in our study. Webb et al. (2013) [19] worked with five RCTs and observational studies. Ito (2014) [15] included four RCTs and observational studies. Francis et al. (2015) [22] used three RCTs, retrospective cohorts, and case series. O'Shea et al. (2017) [10] focused on five RCTs. Hill et al. [20] worked with four RCTs and non‐randomized samples. Visconti et al. [21] included three RCTs, prospective, and longitudinal studies. Shekher et al. [16] analyzed five RCTs in a meta‐analysis, as well as studies on surgery and repair time. Bruney et al. (2022) [13] used four RCTs and non‐randomized studies. Hatami et al. (2022) [23] included three RCTs, case series, prospective studies, descriptive, observational, and retrospective studies. Cordray et al. (2023) [14] worked with four RCTs, pre–post intervention studies, and prospective cross‐sectional studies. Souza et al. (2024) [17] included 46 studies, but only one was an RCT. Power, Murphy et al. (2015) [6] analyzed nine studies on frenotomies: four SRs and five RCTs. Butenko et al. (2019) [7] reviewed studies [10, 19] covering six RCTs, but one of them was a patient over 3 years old (Figure 3).

### 3.3 Breastfeeding‐Related Outcomes

Table 1 provides a summary of the main studies that investigated the effects of frenotomy in infants with ankyloglossia. Detailed information is presented regarding the outcomes—maternal self‐efficacy, nipple pain, and weight gain—as well as the authors' conclusions on the effectiveness of the intervention and the methodological weaknesses identified in each study. This table aims to provide a critical and comparative overview of the available literature, highlighting the results of the outcomes and the limitations that compromise the robustness of the evidence and the clinical applicability of the findings.

**TABLE 1:** Effects of frenotomy in infants with ankyloglossia: Information on the outcomes evaluated (maternal breastfeeding self‐efficacy, nipple pain, and infant weight gain), as well as the main findings and conclusions drawn by the authors concerning the effectiveness of the intervention. The methodological limitations identified in primary studies are also reported.

Author, year	Outcome assessment tools	Outcomes analyzed	Main results	Main conclusions	Methodological weaknesses of the primary studies
Cordray et al. (2023)	BSES‐SF, VAS, weight records	Maternal self‐efficacy, nipple pain, weight gain, gastroesophageal reflux	Significant improvement in self‐efficacy, reduction in nipple pain, weight gain, and improvement in gastroesophageal reflux	Frenotomy was effective for all evaluated outcomes	Heterogeneity among included studies; few randomized controlled trials (RCTs); predominance of subjective outcome measures; lack of control groups for certain outcomes
Finigan et al. (2013)	Weight percentile curves	Maternal breastfeeding experience before and after the intervention; objective measures, such as tongue function assessment; improvement in breastfeeding evaluation using tools, such as LATCH, IBFAT, VAS, and SM‐FPQ	Mean increase of 15 percentiles post‐procedure (*p* < 0.001)	Frenotomy associated with better weight gain	Selection bias, as parental consent decisions may be influenced by external factors. In many cases, the mother had attended a breastfeeding clinic or received guidance from a breastfeeding specialist prior to referral. There may be a preponderance in study samples of mothers who were already more motivated to breastfeed and who had positive expectations regarding the effectiveness of frenotomy
Francis et al. (2015)	Questionários autorreferidos, LATCH, IBFAT, BSES‐SF. Vas Short Form McGill Pan Questionare	Maternal self‐efficacy, nipple pain	Subjective improvement reported by mothers	Possible perceived benefit, but lacking robustness	Small sample; lack of standardization; no validated scales
Ito et al. (2014)	LATCH, IBFAT; VAS, body weight, adverse events	Nipple pain, weight gain	Pain reduction; average gain of 15 centiles in 2 weeks	Positive efficacy for pain and weight	Predominance of non‐randomized studies; risk of bias
Hill et al. (2020)	Maternal reports, observation clínica	Self‐efficacy, pain, weight gain, gastroesophageal reflux	Reported improvement in pain and self‐efficacy; limited data on weight	Indirect evidence of effectiveness; partial benefits	Absence of objective measures and control; short follow‐up, non‐validated instruments
Butenko et al. (2019)	Maternal reports, clinical evaluation	Nipple pain, self‐efficacy	Subjective reduction of pain; partial improvement in self‐efficacy. Limited conclusions in response to the original research question of the effectiveness of a lingual frenotomy in improving breastfeeding abilities	Possible immediate but not sustained benefit. The effectiveness of a frenotomy on breastfeeding abilities when compared to less invasive intervention remains relatively unclear	No control group; subjective measures; lack of follow‐up; non‐validated tools
Shekher et al. (2021)	VAS, BSES‐SF, LATCH, IBFAT, BSES‐SF, VAS, SM‐FPQ.	Nipple pain, self‐efficacy	Reduction in nipple pain, partial improvement in self‐efficacy	Efficacy limited to pain relief	High heterogeneity; absence of blinding in included studies. In addition, few randomized studies on the topic
Souza et al. (2024)	Narratives, qualitative thematic synthesis; maternal experience; Intervening in ankyloglossia and changes in breastfeeding	Pain, self‐efficacy, breastfeeding difficulties	Frenotomia considerada eficaz em contextos específicos. Mos of studies showed that it was effective in reducing negative symptoms during breastfeeding, especially maternal pain and difficulty latching on	Recommended with individualized evaluation	Scoping review; predominance of qualitative studies; only 01 randomized controlled trial
Visconti et al. (2021)	BSES‐SF, VAS, LATCH, IBFAT	Maternal self‐efficacy, nipple pain	Improvement in pain scores and self‐efficacy The classification of ankyloglossia, assessment tools used, age and timing of frenotomy, in terms of breastfeeding improvements were inconsistent across the studies	Frenotomy reduce nipple pain and improve maternal self‐efficacy during breastfeeding. the review also revealed inconsistent definitions of ankyloglossia severity, standardized outcome measures and research protocols	Inconsistent diagnostic criteria; non‐standardized scales; lack of objective measures; variability was noted in the selected measurement tools for each study; absence of controls and randomization in our of the seven studies, which may have contributed to greater bias at the study level
Webb et al. (2013)	IBFAT, LATCH, SF‐MPQ, Milk production and feeding characteristics	Self‐efficacy, pain, weight gain	Improvement in the three subjective outcomes	Limited but favorable evidence. Four studies in the current review used such scale to assess maternal pain and they all demonstrated significant improvements post‐frenotomy. However, only one study had a control group. and therefore, treatment bias may have contributed to the positive results	Low level of evidence; self‐reported measures; treatment bias may have contributed to the positive results
Segal et al. (2007)	Análise descritiva narrativa	Nipple pain, self‐efficacy	Nipple pain, self‐efficacy	Potencial benefício; resultados não conclusivos. there was a limited number of studies available with quality evidence	Studies included without diagnostic standardization; absence of risk of bias analysis of the studies
Power & Murphy (2015)	LATCH scores (3 studies), SF‐MPQ Index (2 studies), IBFAT (1 study), feeding characteristics (3 studies)	Nipple pain, self‐efficacy	Reports of reduced pain in some studies	Evidência sugestiva, porém limitada. subjective maternal improvement in breast feeding in infants with difficulty in breast feeding attributable to tongue‐tie	Based on uncontrolled studies; descriptive analysis; selection bias
Hatami et al. (2022)	U‐TAP, VAS, LATCH and IBFAT, as well as subjective reports	Nipple pain, self‐efficacy	Breastfeeding outcomes were subjectively reported and showed that 35% had mild improvement, followed by 14% with moderate improvement and 7% with marked improvement	Improvements reported, but with variability between tools. lack of consensus regarding pre‐treatment assessment methods, diverse outcome measures, and poor study design and methodology, precise quantification of any improvement cannot be calculated.	No control group; subjective measures; lack of standardization; Significant heterogeneity. Pre‐treatment baselines were mentioned but not reported. “a lack of professional education and general consensus on tools for the assessment and diagnosis of tongue‐tie has led to significant inconsistencies in practice habits. This is confounded by the wide variation in applied surgical techniques, which further complicates obtaining consensus
Bruney et al. (2021)	BSES‐SF, VAS, LATCH, BSES	Breastfeeding self‐efficacy, maternal pain, weight gain	Improvement in breastfeeding difficulties and pain	Effective frenotomy according to evaluated RCTs	Heterogeneity and publication bias; studies with blinding limitations; despite the improvement observed in breastfeeding‐related difficulties and pain, the lack of data on risks may compromise a complete clinical view
O'Shea et al. (2017)	LATCH, IBFAT, VAS, SF‐MPQ, Qualitative assessment of breastfeeding through a questionnaire for parents	Nipple pain, self‐efficacy	Reduction in pain; no improvement in breastfeeding effectiveness	The potential for improving infant breastfeeding and reducing maternal nipple pain may be greater when the diagnosis is severe ankyloglossia. However, only one clinical trial has evaluated frenotomy in this population. Similarly, only one study has investigated frenotomy in infants diagnosed with moderate ankyloglossia and concluded that the procedure had no effect on infant feeding or maternal nipple pain, although it was considered subjectively effective	Low quality of included evidence; small sample size; no study reported breastfeeding duration by group, and all had very high rates of contamination in the control group, which makes long‐term outcomes of little significance. All five studies offered and performed frenotomy in the control group infants, which suggests a lack of equipoise (genuine clinical equipoise). Two studies—Berry (2012) and Dollberg (2006)—performed frenotomy in all participants as part of the study protocol. Buryk (2011), Emond (2013) and Hogan (2005) offered the procedure to controls, with adherence rates ranging from 77% to 97%

Abbreviations: BSES‐SF, Breastfeeding Self‐Efficacy Scale‐Short Form; SF‐MPQ, Short Form McGill Pain Questionnaire; VAS, Pain Visual Analogue Scale; U‐TAP, U‐TAP.

### 3.4 Tools for Measuring Outcomes

The breastfeeding‐related outcome measurement tools used in the primary studies were diverse: (1) the LATCH score (Latch, Audible Swallowing, Type of Nipple, Comfort, Hold), for analyzing breastfeeding efficacy, including latch‐on ability, audible swallowing, nipple type, comfort, and hold [10, 13, 14, 19, 20, 21, 22, 23, 25]; (2) the Infant Breastfeeding Assessment Tool (IBFAT), which subjectively measures the infant's feeding behaviors reported by the mother, related to seeking, stimulus needed, time needed to latch on, feeding patterns, and satisfaction [13, 14, 19, 20, 21, 22, 23, 25, 27, 28]; (3) the Breastfeeding Self‐Efficacy Score‐Short Form (BSES‐SF), which measures breastfeeding efficacy and maternal confidence in their ability to breastfeed [16, 20, 21, 22]; (4) the Bristol Tongue Assessment Tool, which combines parts of the LATCH score with the sucking behavior included in the Infant Breastfeeding Assessment Tool score [20].

The outcome measurement tools used to assess maternal pain during breastfeeding were as follows: (1) the Visual Analog Scale (VAS) or numerical pain scale [10, 13, 14, 15, 16, 19, 20, 21, 22, 23, 25, 26]; and (2) the short form of the McGill Pain Questionnaire [10, 13, 14, 15, 16, 19, 20, 21, 22, 23, 25, 26]. Other outcomes were also considered, such as the duration of breastfeeding in days, the end of breastfeeding according to maternal report, and the infant's pain assessment (Modified Behavioral Pain Scales—MBPS); Neonatal Infant Pain Scale (NIPS) and CRIES Pain Scale (Neonatal Postoperative Pain Measurement Score), which includes crying, need for oxygen, increased vital signs, facial expression, and sleep patterns [10].

Four of the reviews looked at weight gain [13, 14, 15, 16, 19]; two of them [14, 22] also reported on aspects, such as milk production/supply, focusing on milk transfer (mL/min) and the rate of breastfeeding continuity, as well as investigating possible adverse events. Hill et al. [20] and Cordray et al. (2023) [14] included outcomes related to infant gastroesophageal reflux, using the I‐GERQ‐R scale.

### 3.5 Overlapping

Many RCTs overlap in the studies reviewed, resulting in 12 separate RCTs. Buryk et al. [27, 29] and Emond et al. (2014) [30], Berry et al. (2012) [28] most frequently, followed by Berry et al. (2012) [28], Dollberg et al. (2006) [31], and Hogan et al. (2005) [32] Other studies included Kim et al. [33] Yousefi et al. (2015) [34], Ghaheri et al. (2022) [35], Amir et al. (2006) [36] Heller et al. (2005) [37] Griffiths et al. (2004) [38] and Ballard et al. (2002) [39] each analyzed only once. Two studies, Butenko et al. (2019) [7] and Power and Murphy (2015) [6] included in their manuscripts SRs that were also analyzed by us, with overlapping between them of only one (Webb et al. 2013) [19] (Tables 2 and 3).

**TABLE 2:** Overlapping of primary studies included in systematic review.

SR included: Design (number of RCT)	Amir et al. (2006)	Ballard et al. (2002)	Berry et al. (2012)	Buryk et al. (2011)	Dollberg et al. (2006)	Emond et al. (2014)	Ghaheri et al. (2022)	Heller et al. (2005)	Griffiths (2004)	Hogan et al. (2005)	Kim et al. (2020)	Yousefi et al. (2015)
Bruney et al. (2022): RTC (4)			x	x	x	x						
Butenko et al. (2019): RTC (6)			x		x	x		x		x		
Cordray et al. (2023): RTC (4)				x	x	x	x					
Finigan et al. (2013): RTC (5)	x		x	x	x			x		x		
Francis et al. (2015): RTC (5)												
Hatami et al. (2022): RTC (3)				x							x	x
Hill et al. (2020): RTC (4)		x		x		x				x		
Ito (2014): RTC (4)			x	x	x			x		x		
O'Shea et al. (2017): RTC (5)												
Power and Murphy (2015): RTC (5)			x	x	x	x				x		
Segal et al. (2007): RTC (1)									x			
Shekher et al. (2021): RCT (5)			x	x	x	x				x		
Souza et al. (2024): RTC (1)						x						
Visconti et al. (2021): RTC (3)			x	x		x						
Webb et al. (2013): RTC (5)			x	x	x	x				x		

Abbreviations: RCT, randomized controlled trials; SR, systematic reviews [27, 28, 30–39].

**TABLE 3:** Overlapping of systematic review included in overviews included in our study.

Overview/study that reviewed SR (number of SR)	Segal et al. (2007)	Webb, Hao, Hong (2013)	Finigan et al. (2013)	Ito (2014)	Francis et al. (2015)	Otawa et al. (2016)^a^	O'Shea et al. (2017)
Butenko et al. (2019) (3)		x				x	x
Power and Murphy (2015) (4)	x	x	x	x			
Otawa et al. (2016)^a^ (2)					x		x

^a^ Book—not considered in our analysis.

### 3.6 Analysis of Methodological Quality

**FIGURE 4:**
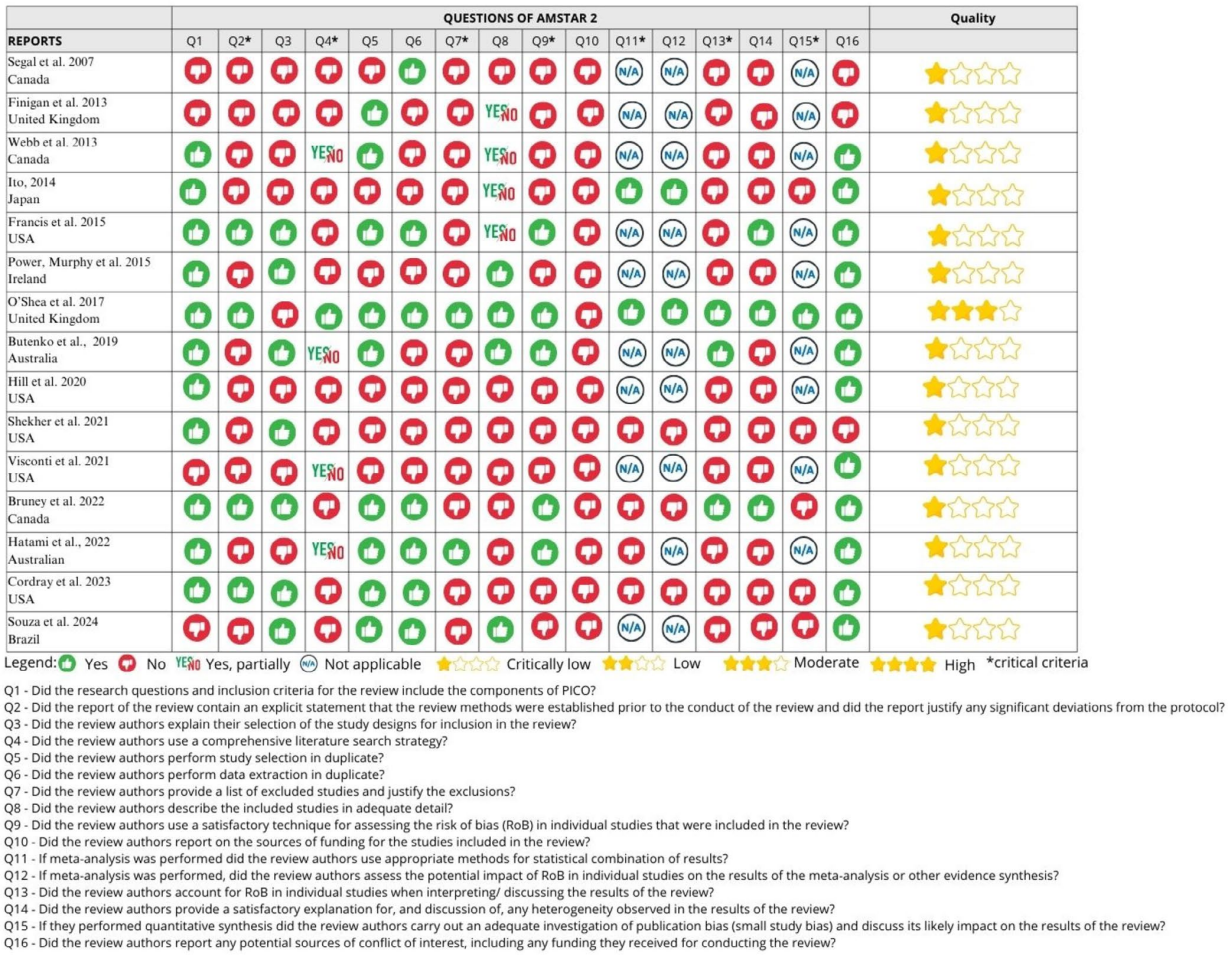
Appraisal methodological quality of systematic reviews using the Amstar 2 [7, 10, 13, 22, 23].

Figure 4 shows an assessment of the methodological quality of the SRs using AMSTAR 2. The overall assessment of the SR results showed that 14 reviews were classified as critically low, with the most failed items being 7 related to the presentation of the list of excluded studies and their respective justifications (*n* = 13; 86.6%), item 10 about the importance of declaring the sources of funding (*n* = 15; 100%), item 13 related to review authors account for RoB in individuals studies when interpreting/discussing the results of the review, and item 14 related to whether the authors of the review provide satisfactory explanations and discussion of any heterogeneity observed in the review results (*n* = 12; 80.0%). One SR was classified as moderate quality due to the lack of information on the funding of the primary studies and the absence of explanation on the selection of study designs for inclusion [10]. The others had more than one critical flaw, with non‐critical weaknesses.

No study reported the sources of funding for the studies included in the review. Four SRs denied that the review methods were defined prior to conducting the review, with the publication of a registered protocol that included the review question, the search strategy, the inclusion/exclusion criteria, the risk of bias, the synthesis plan, and the investigation of sources of heterogeneity [10, 13, 14, 22]. Two studies provided a list of excluded studies, with the reasons for exclusion [10, 23]. Five SRs assessed the risk of bias of the primary studies [7, 10, 13, 22, 23], but only one included a discussion of the impact of the risk of bias on the results of the review [10].

## 4 Discussion

The umbrella review was organized to clearly separate the discussion of clinical findings related to breastfeeding from the evaluation of the methodological rigor of the SR included in the study.

Some studies have reported improvements in maternal self‐efficacy after frenotomy, based on scales, such as the Breastfeeding Self‐Efficacy Scale‐Short Form (BSES‐SF) [14, 21]. However, others found no statistically significant difference [16]. Most studies used self‐reported measures, without a control group or with short‐term evaluations, limiting the external validity of these findings [7, 26]. In addition, few studies performed analysis between evaluators or controlled for expectation bias, which weakens the interpretation of positive results.

The review by O'Shea et al. (2017) [10], which included five studies with a control group (*n* = 302), concluded that although frenotomy provided greater maternal comfort during breastfeeding, it did not solve the baby's sucking problems. The quality of the evidence was considered low, and new studies may alter these conclusions. Regarding maternal pain, a small but statistically significant effect was observed. There is consistency between the studies that indicate a reduction in pain after the procedure [10, 15, 19]; however, the scores used were mostly subjective (VAS, SF‐MPQ), without blinding in most of the trials, which may have potentiated the placebo effect [6, 25]. The heterogeneity of the instruments used also limits comparability between the studies and compromises the robustness of the conclusions.

The results on weight gain are even more heterogeneous. Shekher et al. [16] discussed the contrast between two small primary studies in which they demonstrated that babies who underwent LF before 8 days of age gained significantly more weight than those who underwent LF after 8 days [40], whereas another retrospective study demonstrated an improvement in breastfeeding regardless of whether the procedure took place before or after 30 days of age [41]. However, these studies cannot be considered solid evidence to support LF due to their methodological limitations. Given the small sample size, it is not possible to generalize the results. Other aspects make their findings less reliable, such as time bias, which prevents control of other variables that may influence weight gain in the first days of life [40], selection bias, and difficulties in standardizing breastfeeding assessments [41].

Other reviews [15, 19, 22] included a limited number of studies that evaluated weight gain [15, 22] and found significant gain in newborns 2 weeks after LF, with a relevant increase of 15 percentiles in the growth curve, evidenced by high statistical significance (*p* < 0.0001) [15]. The continuation of breastfeeding [19, 22] after treatment resulted in a 92% improvement in feeding at 3 months, but these rates fell over time, reaching 28% perceived improvement within 12 months. The I‐GERQ‐R was used in one study to assess complementary outcomes, such as reflux symptoms. Although relevant, this instrument was applied in a limited context and without a clear description of its cultural adaptation or validation for the target population.

Although Finigan et al. (2013) [26] and Cordray et al. (2023) [14] indicate better weight gain after frenotomy, other SRs have not demonstrated a significant or sustained clinical effect [17, 21]. In several studies, weight gain was measured without controlling for confounding factors, such as post‐procedure counseling, milk supplementation, or insufficient follow‐up time [19].

Analysis of the instruments used in the studies included in this umbrella review reveals great methodological heterogeneity in the assessment of outcomes related to pain, breastfeeding efficacy, and maternal self‐efficacy. This variability makes direct comparison between studies difficult and compromises the robustness of the available evidence on the effects of LF on infants. The VAS, SF‐MPQ, and the NRS have been widely used to measure maternal nipple pain [10, 13, 14, 15, 16, 19, 20, 21, 22, 23, 25, 26]. However, their subjective and one‐dimensional nature limits the depth of the analysis of the painful experience, especially when applied without blinding or standardization of the evaluation time. In addition, some reviews [20] recognize that the included studies used pain scales that are not validated for measuring nipple pain, which weakens the consistency of the comparative results.

The breastfeeding‐related outcome measurement tools IBFAT [13, 14, 16, 19, 20, 21, 22, 23, 25, 27, 28] and LATCH [10, 13, 14, 19, 20, 21, 22, 23, 25] although commonly used in the clinical evaluation of breastfeeding, these instruments have important limitations. Both instruments depend on the experience of the evaluator, and their reliability can vary when applied by professionals with different levels of training. The Breastfeeding Self‐Efficacy Scale‐Short Form (BSES‐SF) stands out as one of the few validated and psychometrically robust instruments used in the studies reviewed [7, 14, 17, 21]. Composed of items with Likert‐type scale responses, it offers a reliable measure of the mother's perception of her ability to breastfeed. However, its use has been restricted to a few studies, limiting the possibility of a broader quantitative comparison. In many studies, it was unclear whether the application was standardized or whether inter‐rater validation was carried out, which compromises the reproducibility of the findings.

In addition to weight gain as an outcome [13, 15, 16, 19], aspects, such as milk production/supply, focusing on milk transfer (mL/min) and breastfeeding continuation rate, as well as investigating possible adverse events that have also been reported [14, 22].

The literature reviewed indicates possible benefits of frenotomy in three main outcomes: maternal self‐efficacy in breastfeeding, reduction of nipple pain, and infant weight gain. However, the included SR, which analyzed between 4 and 7 RCTs each, reported high heterogeneity between the primary studies in terms of design, diagnostic criteria for ankyloglossia, and assessment tools.

Several primary studies have limitations that compromise the reliability of the evidence in favor of frenotomy. Small sample sizes were observed in studies, such as Dollberg et al. 2006 (*n* = 25) [31]. Hogan et al. 2005 (*n* = 57) [32] Buryk et al. 2011 (*n* = 58) [27] Berry et al. 2012 (*n* = 57) [26, 28] making it difficult to generalize the results. The absence of blinding was identified in Hogan et al. (2005) [32], in which neither the participants nor the professionals were blinded to the intervention, and in Buryk et al. [29] in which only the evaluators were blinded, favoring performance and expectation bias. The follow‐up time of < 1 month, insufficient to assess sustained effects, was recurrent in trials such as Ballard et al. (2002) [39] and Dollberg et al. (2006) [31] in which outcomes were assessed immediately after the intervention or within 2 weeks. In addition, few studies reported data on complications, cost‐effectiveness, or long‐term impact, and this gap is highlighted in reviews, such as Webb et al. (2013) [19] and Souza et al. (2024) [17].

In addition to the diversity of instruments, there is also a lack of uniform criteria for the diagnosis of ankyloglossia and the time of the postintervention assessment, which generates variations in the sensitivity to change and the observed impact of the frenotomy. In some cases, the studies do not specify when the final results were collected, which limits the longitudinal interpretation of the results.

The majority of the SRs were of critically low quality. The two most worrying items in Amstar 2 were as follows: (1) Item 7, related to the presentation of the list of excluded studies and their respective justifications; (2) Item 10, on the importance of declaring the sources of funding. Another critical aspect was the lack of transparency in the design of the studies, without prior registration of the SRs [6, 7, 15, 16, 17, 19, 20, 21, 23, 25, 26]. Only three SRs take into account the risk of bias in individual studies when discussing the results of the review [7, 13, 23]. Protocol registration is essential to anticipate methodological challenges, reduce bias in the selection of studies or results, and ensure transparency and reproducibility. Lack of registration can affect the integrity and reliability of the results. Finigan et al. (2013) [26], Segal et al. (2007) [25] and Visconti et al. [21] did not score for PICO, due to the lack of comparison groups in both the formulation of the research question and the inclusion criteria. When investigating changes in clinical practice, one of the most important steps is to define the clinical question precisely. The included SRs not only failed to score this item but also provided insufficient information on the inclusion and exclusion criteria [15, 19, 21, 23, 26] of primary studies, as pointed out by Hill et al. [20].

A critical analysis of the SRs on LF in infants showed considerable heterogeneity in the results. The statistical heterogeneity of the studies included in the SRs was not properly addressed. This raised concerns about the applicability and interpretation of the results of the meta‐analyses. The absence of sensitivity analyses and assessment of publication bias is also problematic, given the potential risk of affecting the validity and robustness of the conclusions. Cordray (2023) [14] included 21 studies with more than 1500 children and demonstrated large effects on breastfeeding and pain reduction, but without adequate control groups; only four studies were randomized clinical trials (*n* = 237); different study designs were mixed in the meta‐analysis, based on intra‐group analyses, which compromises the validity of the conclusions. It is not possible to attribute the effects directly to the intervention without considering the natural prognosis of the condition.

Note that the updated version of AMSTAR 2 is from 2017, which may affect the authors' perception of methodological quality [12]. Considering that half of the SRs were published before that date, the authors' observations on the studies may not have covered all the criteria provided by the tool. However, the difference between AMSTAR, published in 2009, and AMSTAR 2 is related to non‐randomized clinical trials, and only one SR was published before 2009 [26]. One SR published in 2017 [10] analyzed only RCTs. The other reviews included RCTs, N‐RCTS, and observational studies.

Although SRs are fundamental for professional decision‐making, none of the SRs included in this study showed high methodological quality. Pauletto et al. (2022) [42] also pointed out the low methodological quality of SRs, showing that 60% of these reviews had critically low quality and 27.9% low quality, with only 1% classified as high quality. This scenario emphasizes the urgent need to improve the methodological standards of SRs to ensure that the evidence is solid. Reliable evidence supports appropriate clinical decision‐making, especially in cases that require surgical intervention (i.e., LF) and that present inherent risks, such as malnutrition, hypovolemic shock, acute airway obstruction, abscesses, lingual nerve damage, and ranula with possible irreversible damage to the salivary gland [43, 44].

The limitations of this study included the difficulties in identifying specific data in the SRs. Most of them included different types of studies with varying methodologies and different age groups, implying heterogeneity between the studies. In addition, the variation in the inclusion and exclusion criteria of the primary studies within the SRs made it difficult to directly compare the results. The lack of uniformity in the presentation of outcomes and the lack of age subgroups in some reviews contributed to the heterogeneity of the data, which affected the accurate assessment of the efficacy of LF in infants up to 1 year of age. These methodological variations prevented the study from providing definitive conclusions. This highlights the need for more rigor and standardization in future research on the subject.

It is crucial that future clinical trials and SRs aimed at studying the progression of LF in infants use a rigorous and transparent methodology, choose appropriate outcomes and analysis tools, and promote longitudinal follow‐up. It is suggested that well‐established methodological guidelines be adopted, that the protocol be prospectively registered, and that a full assessment of the risk of bias and heterogeneity be carried out. Transparency regarding funding sources and potential conflicts of interest is also vital to ensure the integrity and reliability of the evidence. These precautions will contribute to providing solid evidence for clinical practice and to improving the safety and well‐being of children. These requirements allow professionals or anyone else, such as parents, to seek reliable information to avoid unnecessary or hasty interventions.

## Author Contributions

B. V. da S. collected the data, acted as a reviewer, and drafted the original manuscript. A. V. B. conducted the writing and final editing of the manuscript, in addition to the review. N. Da N. L. Sá contributed to the curation and review of the manuscript. L. B. O. contributed to the curation and validation of the entire study, and was also responsible for the conceptualization, methodology, visualization, and mediation. L. F. S. contributed to the curation and validation of the entire study, and was also responsible for the conceptualization, methodology, visualization, and mediation. All authors reviewed and approved the final manuscript, agreeing to its submission and assuming responsibility for the content of the article.

## Disclosure

Protocol and registration: The systematic review protocol was registered in the OSF database (OSF 10.17605/OSF.IO/SA76V). This study follows the guidelines of the Preferred Reporting Items for Systematic Reviews and Meta‐Analyses [45]. In addition, the study's report was elaborated according to PRIO‐harms (Preferred Reporting Items for Overview of Systematic Reviews Checklist) [46]. The research does not require approval from the Research Ethics Committee.

## Conflicts of Interest

The authors declare no conflicts of interest.

## Supporting information


**Appendix S1:** Search strategies.

**Appendix S2:** Excluded articles and reasons for exclusion (*n* = 24).

## Data Availability

The data that support the findings of this study are available on request from the corresponding author. The data are not publicly available due to privacy or ethical restrictions.
