# Supplementary material for: Lingual Frenotomy in Breastfeeding Infants: An Umbrella Review
Source: Int J Paediatr Dent. 2025 Sep 24;36(1):90–104. doi: 10.1111/ipd.70031 (PMC12783447; doi:10.1111/ipd.70031)
Supplement: Supplementary file 1 — Appendix S1: Search strategies. [file IPD-36-90-s001.docx]

**APPENDIX 1** Search strategies.

| Data base | Search strategy (December 18^th^, 2024) | Number of references retrieved |
| --- | --- | --- |
| **PubMed/Medline** | ("Infant"[Mesh] OR Infant* OR "Infant, Newborn"[Mesh] OR newborn* OR "new born" OR "new borns" OR neonate* OR neonatology ) AND ("Ankyloglossia"[Mesh] OR Ankyloglossia* OR "tongue tie" OR "tongue ties" OR "Lingual Frenum"[Mesh] OR "lingual frenum" OR "lingual frenulum" OR "lingual frenulums" OR "lingual frenums" OR frenectom* OR frenulectom* OR "frenulum excision" OR "frenulum removal" OR "frenum excision" OR "frenum removal" OR "frenum resection" OR frenotom* OR frenulotom* OR frenoluplast*) AND ("Weight Gain"[Mesh] OR "Weight Gain" OR "Weight Gains" OR "Weight Loss"[Mesh] OR "Weight Loss" OR "Weight Losses" OR "Weight Reduction" OR "Weight Reductions" OR "Breast Feeding"[Mesh] OR "Breast Feeding" OR Breastfeeding OR Breastfed OR "Breast Fed") AND ("Systematic Review"[Publication Type] OR "Systematic Reviews as Topic"[Mesh] OR "systematic review" OR "systematic reviews" OR "systematic literature review" OR "systematic literature reviews" OR "systematic evidence review" OR "systematic evidence reviews" OR "systematic meta-review" OR "systematic meta reviews" OR "systematic critical review" OR "systematic critical reviews" OR "cochrane review" OR "cochrane reviews" OR "Meta-Analysis" [Publication Type] OR "Meta-Analysis" OR "Metaanalysis" OR "Metanalysis" OR "Meta-Analyses" OR "Metaanalyses" OR "Metanalyses" OR overview* OR "scoping review" OR "scoping reviews") | **30** |
| **Embase** | ('infant'/exp OR infant* OR 'infant, newborn'/exp OR newborn* OR 'new born'/exp OR 'new born' OR 'new borns' OR neonate* OR 'neonatology'/exp OR 'neonatology') AND ( 'ankyloglossia'/exp OR ankyloglossia* OR 'tongue tie'/exp OR 'tongue tie' OR 'tongue ties' OR 'lingual frenum'/exp OR 'lingual frenum' OR 'lingual frenulums' OR 'lingual frenums' OR frenectom* OR frenulectom* OR 'frenulum excision'/exp OR 'frenulum excision' OR 'frenulum removal'/exp OR 'frenulum removal' OR 'frenum excision'/exp OR 'frenum excision' OR 'frenum removal'/exp OR 'frenum removal' OR 'frenum resection'/exp OR 'frenum resection' OR frenotom* OR frenulotom* OR frenoluplast*) AND ('weight gain'/exp OR 'weight gain' OR 'weight gains' OR 'weight loss'/exp OR 'weight loss' OR 'weight losses' OR 'weight reduction'/exp OR 'weight reduction' OR 'weight reductions' OR 'breast feeding'/exp OR 'breast feeding' OR 'breastfeeding'/exp OR breastfeeding OR breastfed OR 'breast fed') AND ('systematic review'/exp OR 'systematic review' OR 'systematic reviews'/exp OR 'systematic reviews' OR 'systematic literature review' OR 'systematic literature reviews' OR 'systematic evidence review' OR 'systematic evidence reviews' OR 'systematic meta-review' OR 'systematic meta reviews' OR 'systematic critical review' OR 'systematic critical reviews' OR 'cochrane review' OR 'cochrane reviews' OR 'meta-analysis'/exp OR 'meta-analysis' OR 'metaanalysis'/exp OR 'metaanalysis' OR 'metanalysis' OR 'meta-analyses' OR 'metaanalyses'/exp OR 'metaanalyses' OR 'metanalyses' OR overview* OR 'scoping review'/exp OR 'scoping review' OR 'scoping reviews') | **47** |
| **Scopus** | TITLE-ABS-KEY(Infant* OR newborn* OR "new born" OR "new borns" OR neonate* OR neonatology) AND TITLE-ABS-KEY(Ankyloglossia* OR "tongue tie" OR "tongue ties" OR "lingual frenum" OR "lingual frenulums" OR "lingual frenums" OR frenectom* OR frenulectom* OR "frenulum excision" OR "frenulum removal" OR "frenum excision" OR "frenum removal" OR "frenum resection" OR frenotom* OR frenulotom* OR frenoluplast*) AND TITLE-ABS-KEY("Weight Gain" OR "Weight Gains" OR "Weight Loss" OR "Weight Losses" OR "Weight Reduction" OR "Weight Reductions" OR "Breast Feeding" OR Breastfeeding OR Breastfed OR "Breast Fed") AND TITLE-ABS-KEY("systematic review" OR "systematic reviews" OR "systematic literature review" OR "systematic literature reviews" OR "systematic evidence review" OR "systematic evidence reviews" OR "systematic meta-review" OR "systematic meta reviews" OR "systematic critical review" OR "systematic critical reviews" OR "cochrane review" OR "cochrane reviews" OR "Meta-Analysis" OR "Metaanalysis" OR "Metanalysis" OR "Meta-Analyses" OR "Metaanalyses" OR "Metanalyses" OR overview* OR "scoping review" OR "scoping reviews") | **41** |
| **Web of Science** | TS=(Infant* OR newborn* OR "new born" OR "new borns" OR neonate* OR neonatology) AND TS=(Ankyloglossia* OR "tongue tie" OR "tongue ties" OR "lingual frenum" OR "lingual frenulums" OR "lingual frenums" OR frenectom* OR frenulectom* OR "frenulum excision" OR "frenulum removal" OR "frenum excision" OR "frenum removal" OR "frenum resection" OR frenotom* OR frenulotom* OR frenoluplast*) AND TS=("Weight Gain" OR "Weight Gains" OR "Weight Loss" OR "Weight Losses" OR "Weight Reduction" OR "Weight Reductions" OR "Breast Feeding" OR Breastfeeding OR Breastfed OR "Breast Fed") AND TS=("systematic review" OR "systematic reviews" OR "systematic literature review" OR "systematic literature reviews" OR "systematic evidence review" OR "systematic evidence reviews" OR "systematic meta-review" OR "systematic meta reviews" OR "systematic critical review" OR "systematic critical reviews" OR "cochrane review" OR "cochrane reviews" OR "Meta-Analysis" OR "Metaanalysis" OR "Metanalysis" OR "Meta-Analyses" OR "Metaanalyses" OR "Metanalyses" OR overview* OR "scoping review" OR "scoping reviews") | **33** |
| **Cochrane** | ("Infant" OR Infant* OR "Infant, Newborn" OR newborn* OR "new born" OR "new borns" OR neonate* OR neonatology ) AND ("Ankyloglossia" OR Ankyloglossia* OR "tongue tie" OR "tongue ties" OR "lingual frenum" OR "lingual frenulums" OR "lingual frenums" OR frenectom* OR frenulectom* OR "frenulum excision" OR "frenulum removal" OR "frenum excision" OR "frenum removal" OR "frenum resection" OR frenotom* OR frenulotom* OR frenoluplast*) AND ("Weight Gain" OR "Weight Gains" OR "Weight Loss" OR "Weight Losses" OR "Weight Reduction" OR "Weight Reductions" OR "Breast Feeding" OR Breastfeeding OR Breastfed OR "Breast Fed") | **1** |
| **LILACS** | (infant* OR newborn* OR "new born" OR "new borns" OR neonat* OR lactente*or "Recém nascido" OR "recém nascidos" OR "recém nascida" OR "recém nascidas" OR "Recién Nacido" OR "Recién Nacidos") AND (ankyloglossia* OR "tongue tie" OR "tongue ties" OR "lingual frenum" OR "lingual frenulums" OR "lingual frenums" OR frenectom* OR frenulectom* OR "frenulum excision" OR "frenulum removal" OR "frenum excision" OR "frenum removal" OR "frenum resection" OR frenotom* OR frenulotom* OR frenoluplast* OR anquiloglossia OR "Língua Presa" OR "Freio Lingual" OR "frênulo lingual" OR frenectomia OR frenotomia OR frenulotomia OR frenoluplastia OR anquiloglosia OR "frenillo lingual"or "lengua amarrada" OR "lengua anclada" OR "lengua anudada" OR "lengua atada" OR "frenillo de la lengua" ) AND ("Weight Gain" OR "Weight Gains" OR "Weight Loss" OR "Weight Losses" OR "Weight Reduction" OR "Weight Reductions" OR "Breast Feeding" OR breastfeeding OR breastfed OR "Breast Fed" OR "Aumento de Peso" OR "Ganho de Peso" OR "Redução de Peso" OR "Perda de Peso" OR emagrecimento OR "Ganancia de peso" OR "Perdida de Peso" OR "Reducción de Peso" OR adelgazamiento) AND ("systematic review" OR "systematic reviews" OR "systematic literature review" OR "systematic literature reviews" OR "systematic evidence review" OR "systematic evidence reviews" OR "systematic meta-review" OR "systematic meta reviews" OR "systematic critical review" OR "systematic critical reviews" OR "cochrane review" OR "cochrane reviews" OR "Meta-Analysis" OR "Metaanalysis" OR "Metanalysis" OR "Meta-Analyses" OR "Metaanalyses" OR "Metanalyses" OR overview* OR "scoping review" OR "scoping reviews" OR "Revisão Sistemática" OR "Revisoes Sistemáticas" OR "revisao critica Sistematica" OR "Evidencia Sistematica" OR metanálise* OR "meta analise" OR "meta analises" OR "Revision Sistemática" OR "Revisiónes Sistemáticas" OR metaanalisi* OR "Meta-Análisis" OR "Metanalisis") AND ( db:("LILACS")) | **78** |
| Google Scholar | “infant newborn” AND ankyloglossia AND “systematic review” | **42** |
| **TOTAL** | | **272** |
