# Supplementary material for: Lingual Frenotomy in Breastfeeding Infants: An Umbrella Review
Source: Int J Paediatr Dent. 2025 Sep 24;36(1):90–104. doi: 10.1111/ipd.70031 (PMC12783447; doi:10.1111/ipd.70031)
Supplement: Supplementary file 2 — Appendix S2: Excluded articles and reasons for exclusion (n = 24). [file IPD-36-90-s002.docx]

**APPENDIX 2** Excluded articles and reasons for exclusion (n=24).

| **Author, Year** | **Reason for exclusion** |
| --- | --- |
| 1. Arena et al. (2022) | 1 |
| 1. Carnino et al. (2023) | 5 |
| 1. Chinnadurai et al (2015) | 4 |
| 1. Chetwynd (2024) | 5 |
| 1. Chowdhury et al. (2024) | 4 |
| 1. Constantine et al. (2011) | 2 |
| 1. Cordray et al. (2023) | 4 |
| 1. Francis et al. (2015) | 6 |
| 1. Gonazález Garrido et al. (2022) | 1 |
| 1. Hill, Lee, Pados (2021) | 4 |
| 1. Hill et al., (2019) | 4 |
| 1. Khan et al (2020) | 4 |
| 1. Lawson (2024) | 2 |
| 1. Llanos-Redondo et al (2021) | 5 |
| 1. Miranda et al (2016) | 4 |
| 1. O'Shea et al (2014) | 2 |
| 1. Power and Murphy (2014) | 5 |
| 1. Santos et al (2023) | 5 |
| 1. Shay et al (2016) | 5 |
| 1. Shekher et al. (2019) | 2 |
| 1. Yu et al (2022) | 3 |
| 1. Zander et al (2023a) | 5 |
| 1. Zander et al (2023b) | 3 |
| 1. Zander et al (2023c) | 5 |

Legend: 1- Population over 1 year old (n =2); 2-Abstract (n=4); 3 - Protocol of systematic review (n=2); 4 - Article outside the scope of the study (n=7); 5 - Study design (n=8); 6 - Study repeated (n=1)

**REFERENCES**

1. Arena M, Micarelli A, Guzzo F, et al. Outcomes of tongue-tie release by means of tongue and frenulum assessment tools: a scoping review on non-infants. Acta Otorhinolaryngol Ital. 2022;42(6):492-501.
2. Carnino JM, Walia AS, Lara FR, et al. The effect of frenectomy for tongue-tie, lip-tie, or cheek-tie on breastfeeding outcomes: A systematic review of articles over time and suggestions for management. International Journal of Pediatric Otorhinolaryngology. 2023;111638.
3. Chinnadurai S, Francis DO, Epstein RA, et al. Treatment of ankyloglossia for reasons other than breastfeeding: a systematic review. Pediatrics. 2015;135(6).
4. Chetwynd E, Demirci J. The Gap Between Breastfeeding Research and the Clinical Needs of Lactation Support Providers. J Hum Lact. 2024 May;40(2):195-196.
5. Chowdhury, R., Khoury, S., Leroux, J., Alsayegh, R., Lawlor, C.M., Graham, M.E. Alternative Therapies for Ankyloglossia-Associated Breastfeeding Challenges: A Systematic Review Breastfeeding Medicine. 2024;19(7): 497-504.
6. Constantine AH, Williams C, Sutcliffe AG. A systematic review of frenotomy for ankyloglossia (tongue tie) in breast fed infants. Archives of Disease in Childhood. 2011;96(Suppl 1):A62-A63.
7. Cordray H, Mahendran GN, Tey CS, Nemeth J, Sutcliffe A, Ingram J, Raol N. Severity and prevalence of ankyloglossia-associated breastfeeding symptoms: A systematic review and meta-analysis. Acta Paediatr. 2023 Mar;112(3):347-357.
8. Francis DO, Chinnadurai S, Morad A, et al. Treatments for Ankyloglossia and Ankyloglossia With Concomitant Lip-Tie [Internet]. Rockville (MD): Agency for Healthcare Research and Quality (US); 2015. Report No.: 15-EHC011-EF.
9. González Garrido MDP, Garcia-Munoz C, Rodríguez-Huguet M, et al. Effectiveness of Myofunctional Therapy in Ankyloglossia: A Systematic Review. International Journal of Environmental Research and Public Health. 2022;19(19):12347.
10. Hill, R DNP, MSN, RN, FNP, CNE. Implications of Ankyloglossia on Breastfeeding. MCN, The American Journal of Maternal/Child Nursing 44(2):p 73-79, March/April 2019.
11. Hill RR, Lee CS, Pados B.F. The prevalence of ankyloglossia in children aged <1 year: a systematic review and meta-analysis. Pediatr Res. 2021(90):259–266.
12. Khan U, MacPherson J, Bezuhly M, et al. Comparison of Frenotomy Techniques for the Treatment of Ankyloglossia in Children: A Systematic Review. Otolaryngol Head Neck Surg. 2020;163(3):428-443.
13. Lawson, H., Evans, L., Knights, F., Chadha, K., Oakeshott, P. Frenulectomy as management for tongue-tie in breastfeeding problems: a rapid review. Arch. Dis. Child. 2024; 109(0):A52-A53.
14. Llanos-Redondo A, Contreras-Suárez K, Aguilar-Cañas SJ. Evaluación del frenillo lingual en neonatos. Una revisión sistemática Evaluation of the lingual frenulum in neonates. A Systematic Review. Revista Investigación e innovación en ciencias de la salud. 2021;3(1).
15. Miranda PP, Cardoso CL, Gomes E. Interventions in the Alteration on Lingual Frenum: Systematic Review. International Archives of Otorhinolaryngology. 2016;20:275-280.
16. O'Shea JE, Foster JP, Jacobs SE, et al. Frenotomy for tongue-tie in newborn infants. Paediatr. Child Health. 2014;50:25.
17. Power RF, Murphy JF. Tongue-tie and frenotomy in infants with breastfeeding difficulties: achieving a balance. Arch Dis Child. 2014;100(5):489-494. doi: 10.1136/archdischild-2014-306211. Epub 2014 Nov 7. PMID: 25381293.
18. Santos HKMPDS, Cunha DAD, Andrade RAD, et al. Effects of lingual frenotomy on breastfeeding and electrical activity of the masseter and suprahyoid muscles. CoDAS. 2023;35
19. Shay S, Mandelbaum R, Shapiro N. Tongue tie in infancy. Current Treatment Options in Pediatrics. 2016;2:246-255.
20. Shekher R, Hoppe I, Taylor J, et al. When should we perform frenulectomy for tongue-tie? An evidence-based algorithm of care. ACPA’s 76th Annual Meeting Abstracts. The Cleft Palate-craniofacial Journal. 2019;56(Suppl 1):1-130.
21. Yu C, Li QQ, Zhang RZ, et al. Protocol: Effects of different surgical treatments on children with ankyloglossia: protocol for a systematic review and meta-analysis. BMJ open. 2022;12(12).
22. Zander LRM, do Amaral I, Fadel CB, et al. Anquiloglossia e frenotomia lingual em neonatos: revisão de escopo. OBSERVATÓRIO DE LA ECONOMÍA LATINOAMERICANA. 2023a;21(4):2014-2048.
23. Zander LRM, Kluthcovsky ACGC, do Amaral I, et al. Anquiloglossia e frenotomia lingual em neonatos: protocolo de revisão de escopo. Research, Society and Development. 2023b;12(1)
24. Zander LRM. Fatores associados ao padrão de aleitamento em neonatos submetidos à frenotomia lingual. Dissertação (Mestrado em Ciências da Saúde - Área de concentração: Atenção Interdisciplinar em Saúde), Universidade Estadual de Ponta Grossa. 2023c.
